# Avoided Crossing Phonons Realizes High‐Performance Single‐Crystalline *β*‐Zn_4_Sb_3_ Thermoelectrics

**DOI:** 10.1002/advs.202411498

**Published:** 2024-12-11

**Authors:** I‐Lun Jen, Cheng‐Yen Lin, Kuang‐Kuo Wang, Chun‐Ming Wu, Chi‐Hung Lee, Hsin‐Jay Wu

**Affiliations:** ^1^ Department of Materials Science and Engineering National Taiwan University Taipei 10617 Taiwan; ^2^ Department of Materials Science and Engineering National Yang Ming Chiao Tung University Hsinchu 30010 Taiwan; ^3^ Department of Applied Physics Tunghai University Taichung 407224 Taiwan; ^4^ Department of Materials and Optoelectronic Science National Sun Yat‐sen University Kaohsiung 80424 Taiwan; ^5^ National Synchrotron Radiation Research Center Hsinchu 300092 Taiwan

**Keywords:** avoid crossing phonon, conversion efficiency, moiré fringes, thermoelectrics, β‐Zn_4_Sb_3_

## Abstract

This study reveals the mechanisms behind the ultralow lattice thermal conductivity *κ*_L_ in *β*‐Zn_4_Sb_3_ single crystals through inelastic neutron scattering (INS). Analyzing phonon behaviors and the interaction between acoustic phonons and rattling modes, the first experimental evidence of avoided crossing in *β*‐Zn_4_Sb_3_ is provided. The rattler‐phonon avoided crossings contribute to the low *κ*_L_ in a *β*‐Zn_4_Sb_3_ single crystal, enhancing the thermoelectric figure‐of‐merit (*zT*). TEM characterizations of the *β*‐Zn_4_Sb_3_ single crystal with intrinsic and ultralow *κ*_L_ reveal a grain‐boundary‐free structure with uniformly dispersed rotation moiré fringes that contribute to low lattice thermal conductivity while maintaining a uniform elemental distribution. Additionally, the significant impact of crystallinity control coupled with dilute doping on boosting thermoelectric performance, with single‐crystalline single leg outperforming their polycrystalline counterparts is demonstrated. Notably, the conversion efficiency *η* of the undoped *β*‐Zn_4_Sb_3_ single leg achieves 1.4% under a temperature gradient of 200 K.

## Graphical abstract

This research uncovers the origins of ultralow lattice thermal conductivity in *β*‐Zn₄Sb₃, presenting the first experimental proof of avoided crossings between acoustic phonons and rattling modes via inelastic neutron scattering while demonstrating how crystallinity control significantly enhances thermoelectric performance, leading to a promising conversion efficiency of 1.6% in single‐crystalline *β*‐Zn₄Sb₃ single‐leg under a 175 K temperature gradient.

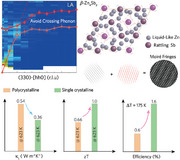

## 1 Introduction

Developing high‐performance green energy requires high production rates, environmental friendliness, and cost‐effectiveness. These criteria are essential to ensure that green energy is as effective as fossil fuels. Thermoelectric (TE) materials, which convert waste heat to electricity via solid‐state reactions,^[^ ^1^ ^]^ are ideal candidates. Their performance is governed by a dimensionless figure‐of‐merit *zT* = *S* ^2^𝜎*κ* ^−1^T, where *S* is the Seebeck coefficient, *σ* is the electrical conductivity, and 𝜅 is the thermal conductivity. As the *zT* increases, the conversion efficiency $\eta =\left(\frac{T_{h}-T_{c}}{T_{h}}\right)\frac{\sqrt{1+zT_{ave}}-1}{\sqrt{1+zT_{ave}}+T_{C}/T_{H}}$ is proportionally enhanced.

Among all the TE materials, zinc antimonides are the pioneering and the only tellurium‐free option in thermoelectric generator (TEG) development history.^[^ ^2^ ^]^ In particular, the *p*‐type *β*‐Zn_4_Sb_3_, which crystallizes in a hexagonal rhombohedral structure with an R $\bar{3}$ c space group garners significant attention due to its promising *zT* value even without doping. Detailed structural analysis^[^ ^3^ ^]^ suggests that *β*‐Zn_4_Sb_3_, classified as a phonon‐glass electron‐crystal (PGEC) solid, exhibits significant disorder with Zn atoms occupying various defect positions. Specifically, the presence of Zn interstitials, which hop from one interstitial site to another as rapid diffusion species, as predicted by previous DFT calculations, accounts for the inherently low lattice thermal conductivity (*κ*_L_) of *β*‐Zn₄Sb₃.^[^ ^4^ ^]^

Furthermore, an annealing‐diffusion experiment utilizing a zinc isotope as a tracer determined an exceptionally high diffusion coefficient of 10^−5^ cm^2^ s^−1^,^[^ ^5^ ^]^ further confirming that the diffusion behavior of zinc interstitials is analogous to that of liquid‐like species. In contrast, Sb atoms form a rhombohedral framework and act as rattlers, with the rattling of Sb₂ dumbbells contributing to a flat mode in phonon dispersion.^[^ ^6^ ^]^ Additionally, avoided crossing in phonon dispersion, theoretically predicted in many host–guest crystalline materials such as skutterudites, clathrates, AgIn₅S₈, YbFe₄Sb₁₂, and Co‐doped Zr₃Ni₃Sb₄,^[^ ^7^, ^8^, ^9^ ^]^ could be a key mechanism underlying the ultralow *κ*_L_ in these crystalline solids.^[^ ^10^, ^11^, ^12^, ^13^, ^14^ ^]^ However, experimental evidence for avoided crossings is scarce,^[^ ^10^, ^11^, ^15^ ^]^ primarily due to measurement limitations or the flat mode energy is not sufficiently low.^[^ ^16^ ^]^ Only inelastic neutron scattering (INS) has the energy and momentum resolution to detect avoided crossings. It is important to note that the INS measurements using polycrystalline samples only detect the density of states, whereas single crystals are required to distinguish the directions of phonon wavevectors.

To date, avoided crossing in *β*‐Zn₄Sb₃ has not yet been reported, either experimentally or theoretically. In this study, we use single‐crystal INS measurements to demonstrate avoided crossing in the *β*‐Zn₄Sb₃ lattice, revealing the mechanism behind its ultralow *κ*_L_ in this PGEC solid. Beyond its glass‐like *κ*_L_, *β*‐Zn₄Sb₃ has an electronic structure similar to a degenerate semiconductor with a small bandgap of 0.8 eV. Its electrical conductivity can be significantly enhanced through dilute doping, as seen in Ga‐Zn₄Sb₃^[^ ^17^ ^]^ and Al‐Zn₄Sb₃.^[^ ^18^ ^]^ As a result, *β*‐Zn₄Sb₃ exhibits a higher degree of disorder than fully crystalline, defect‐free solids while maintaining excellent electrical conductivity due to effective electron conduction pathways.^[^ ^1^ ^]^ This PGEC characteristic gives *β*‐Zn₄Sb₃ high tunability, enabling exceptional TE performance through *PF*‐*κ* decoupling.^[^ ^19^ ^]^

## 2 Results and Discussion

**Figure 1:**
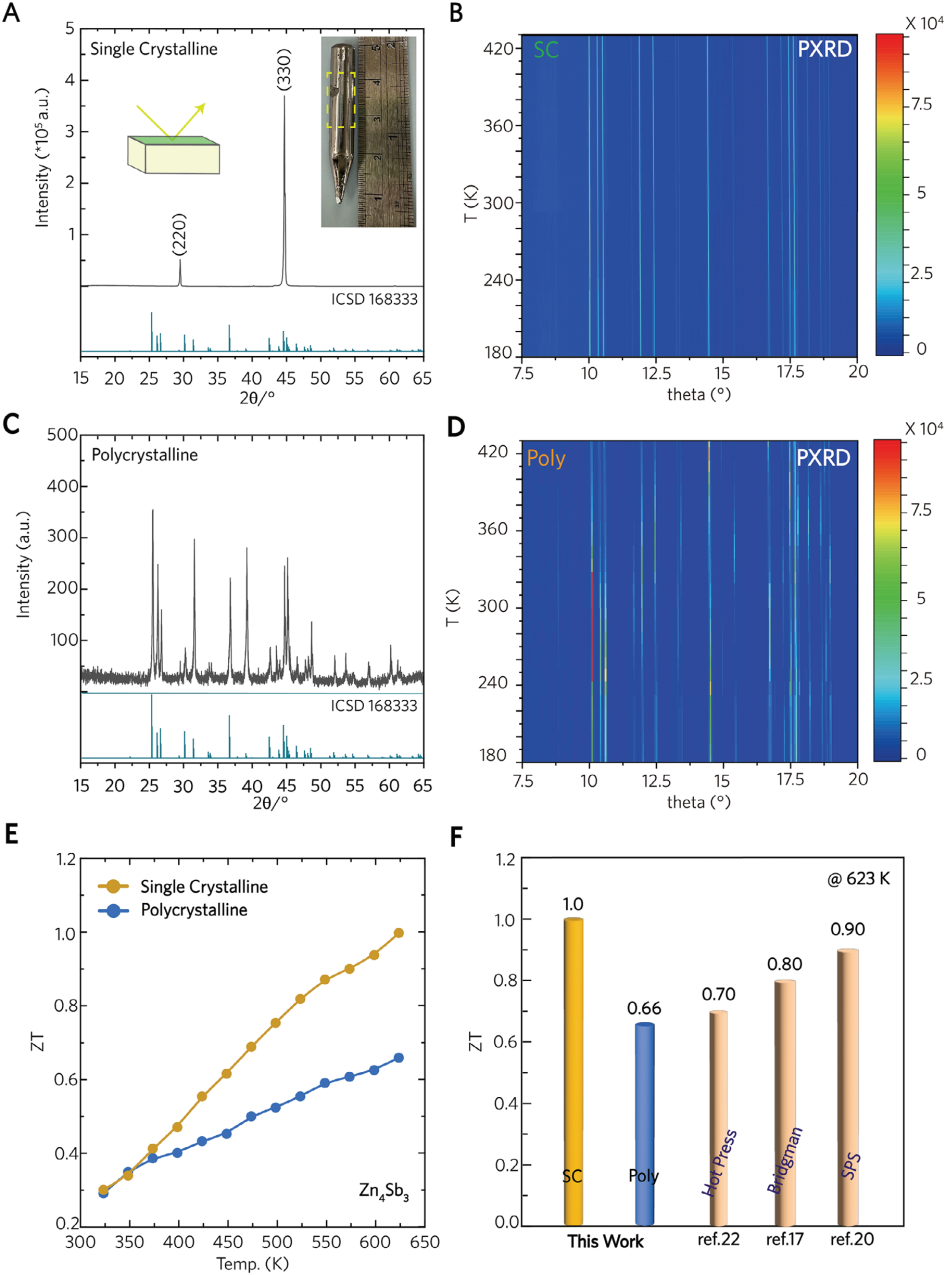
Bulk XRD pattern at room temperature and powder XRD pattern obtained synchrotron radiation source within 180–420 K, for A), B) single crystalline Zn_4_Sb_3_ and for C), D) poly‐crystalline Zn_4_Sb_3_. The inset in (A) shows the as‐growth *β*‐Zn_4_Sb_3_ single crystal. E) The figure of merit *zT* versus temperature. F) Comparison of *zT* values between this work and the previous reports.

Two samples were prepared according to the equilibrium composition of single‐phase *β*‐Zn_4_Sb_3_ under different synthesis conditions. One sample was synthesized using the Bridgman growth process, while the other was furnace‐cooled. Their microstructures are shown in Figure S1 (Supporting Information). The XRD patterns, collected in bulk form along the axial direction of both samples, clearly perform distinct degrees of structural ordering. The XRD pattern of the Bridgman‐grown sample, shown in **Figure** **1**A, exhibits characteristic peaks corresponding to the (220) and (330) crystallographic planes, confirming its single‐crystalline nature (denoted as SC). In contrast, the XRD pattern of the furnace‐cooled sample, depicted in Figure 1C, presents a typical polycrystalline pattern of *β*‐Zn₄Sb₃ (denoted as Poly), with the inevitable formation of secondary ZnSb and Zn phases. In addition to the bulk XRD patterns, the synchrotron‐radiation powder XRD pattern (Figure 1B) confirms that the SC sample is a single‐phase *β*‐Zn₄Sb₃ across a broad temperature range (180–420 K). Meanwhile, additional diffraction peaks were identified in the SC sample (Figure 1D), signifying the presence of impurity phases.

Although the two samples exhibit different crystallinity and distinct phase purity, their *zT* values (Figure 1E) are similar at ambient temperatures; however, their *zT* curves start to diverge significantly when the temperature exceeds 350 K. The *zT* curve of the SC sample outperforms that of the Poly one, reaching a maximum *zT* value of 1.0 at 623 K. The variation in *zT* values between the SC and Poly samples is consistent with previous studies, which report that different synthesis methods significantly influence the thermoelectric performance of *β*‐Zn₄Sb₃.^[^ ^17^, ^18^, ^19^, ^20^ ^]^ Figure 1E shows the diverging *zT* curves of the SC and Poly samples as the temperature increases. Compared with previous studies on *β*‐Zn₄Sb₃,^[^ ^17^, ^18^, ^19^, ^21^ ^]^ Figure 1F indicates that the *zT* value for undoped *β*‐Zn₄Sb₃ ranges from 0.6 to 1.0 at 623 K. In this context, the underlying causes of the varying thermoelectric performance of *β*‐Zn₄Sb₃ with different degrees of crystallinity will be discussed.

**Figure 2:**
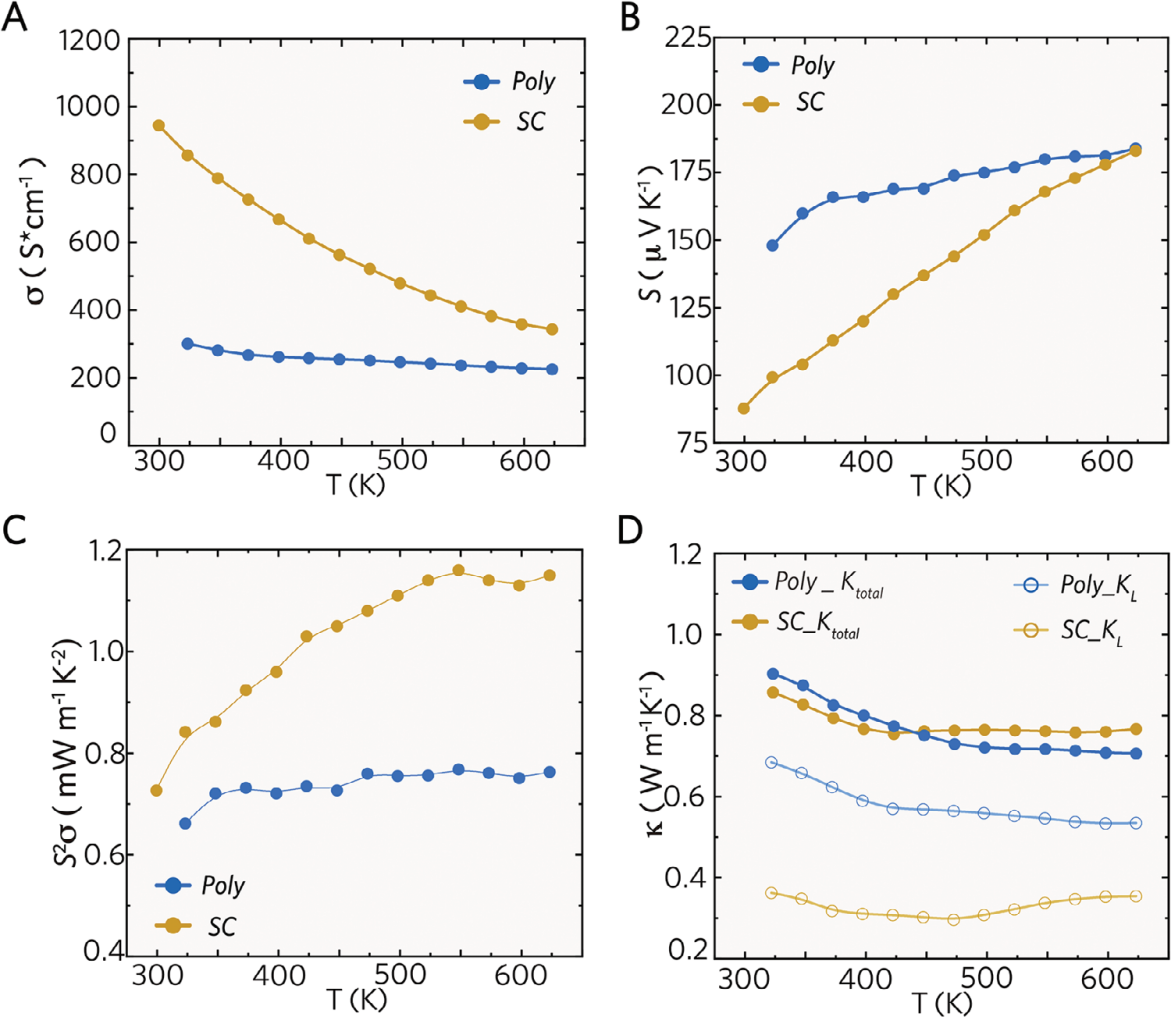
The temperature‐dependent A) Electrical Conductivity *σ* for the Zn_4_Sb_3_ alloys, which are single crystal and polycrystalline. B) Seebeck coefficient *S*, C) Power factor *S* ^2^ *σ*, and D) Total/Lattice thermal conductivity *κ*/*κ_L_* from 300 to 623 K.

The SC and Poly samples exhibit distinct electrical transport properties, with the *σ* curve of the SC sample outperforming that of the Poly sample (**Figure** **2**A). The higher *σ* of the SC sample can be attributed to its larger mobility *µ*_H_ of 65.2 cm^2^V^−1^s^−1^, as indicated in **Table** **1**. This is likely due to the lower defect density in the SC sample, which results in a reduced scattering. Not only is the *µ*_H_ increased, but the carrier concentration *n*_H_ of the SC sample also remains at an optimal level of 10^20^ cm^−3^ due to the single‐phase feature, similar to what had been reported for an intrinsic *β*‐Zn_4_Sb_3_.^[^ ^22^ ^]^ In contrast, the Poly sample exhibits the inevitable secondary phases and shows a lower *n*_H_ with a decreased *µ*_H_, explaining the degradation *σ*. The *S* values of the two samples, as shown in Figure 2B, therefore perform distinct values, especially at mid‐to‐low temperature regions. However, as the temperature rises to 600 K, the *S* curves converge at ≈175 µV K^−1^, while the *σ* values still differ by a factor of 1.5. The SC sample exhibits higher *σ* than the Poly sample above 600 K, likely due to a larger *µ*_H_ at elevated temperatures resulting from reduced scattering. The convergence of *S* values for both samples indicates the intrinsic *n*_H_ of *β*‐Zn₄Sb₃. The decoupling between *S* and *σ*, especially at the mid‐temperature range, is significant for a larger *PF* of the SC sample, as observed in Figure 2C.

**Table 1:** Thermal and electrical transport properties of single crystalline (SC) and polycrystalline (Poly) Zn_4_Sb_3_.

Sample	*ρ* [m Ω cm]	*S* [µVK^−1^]	κ [Wm^−1^K^−1^]	*zT*peak	*µ*H [cm^2^V^−1^s^−1^]	*n*H [10^19^cm^−3^]
SC	1.06	87.7	0.90	1.00	65.2	9.89
Poly	3.33	148	0.86	0.6	36.0	5.15

Controlling crystallinity significantly affects the thermal transport properties. Figure 2D illustrates the *κ* and *κ*_L_ curves for two samples. While both samples exhibit similar *κ* values, the SC sample demonstrates a much lower *κ*_L_ than the Poly one. An ultralow *κ*_L_ of 0.36 W m^−1^ K^−1^ was found over a wide temperature range for the SC sample, which is crucial for achieving an enhanced peak *zT* of unity in the SC sample. The *κ*_L_ shown in Figure 2D displayed notable differences despite the identical chemical composition of the SC and Poly samples. Most importantly, the SC sample exhibits an ultralow *κ*_L_ below 0.4 W m^−1^ K^−1^ within 300–600 K. Such a low‐lying *κ*_L_ benefits an improved *zT* in the middle‐temperature range for an intrinsic *β*‐Zn_4_Sb_3_ without doping. In contrast, the Poly sample shows a higher *κ*_L_ of 0.7–0.5 W m^−1^ K^−1^ over the same range. Although grain boundaries in the Poly sample could increase phonon scattering, the presence of ZnSb and Zn secondary phases, as indicated by the PXRD pattern (Figure S2, Supporting Information), raises its *κ*_L_. These phases, having higher *κ*_L_ than *β*‐Zn_4_Sb_3_, contribute to the elevated thermal conductivity. Prior studies^[^ ^23^ ^]^ suggest that ZnSb and Zn formation is nearly inevitable during the heating of *β*‐Zn_4_Sb_3_. Thus, our SC sample offers insights into intrinsic *β*‐Zn_4_Sb_3_ without secondary phases or grain boundary effects.

As known, the *κ*_L_ can be theoretically expressed through the Equation (1):

(1) $$\kappa_{L}=\sum_{j}\frac{1}{3}C_{j}v_{j}^{2}\tau_{j}\;$$

where *C*_j_ is the specific heat, *v*_j_ is the phonon group velocity, and *τ*_j_ is the phonon lifetime of phonon mode *j*, respectively. Hence, increasing phonon scattering, reducing *v*_j,_ and shortening *τ*_j_ are effective approaches for lowering the *κ*_L._ As aforementioned, avoiding crossing between acoustic phonons and flat modes is crucial for achieving ultralow thermal conductivity. However, so far, no experimental evidence has been reported for *β*‐Zn_4_Sb_3_.

When one computes the *C*_j_, the Einstein model also needs to be considered in addition to the Debye model. This indicates the presence of non‐dispersive Einstein oscillators. The rattling of Sb_2_ dumbbells may be a candidate for these Einstein oscillators, resulting in a low‐energy flat phonon mode.^[^ ^6^ ^]^ Additionally, as shown in the *κ*_L_ versus mean sound velocity,^[^ ^11^ ^]^ *β*‐Zn₄Sb₃, with a *κ*_L_ of 0.4 W m·K^−1^ and a mean sound velocity of ≈2070 m s^−1^,^[^ ^24^ ^]^ falls within the region suggestive of rattling behavior or the avoided crossing feature.

**Figure 3:**
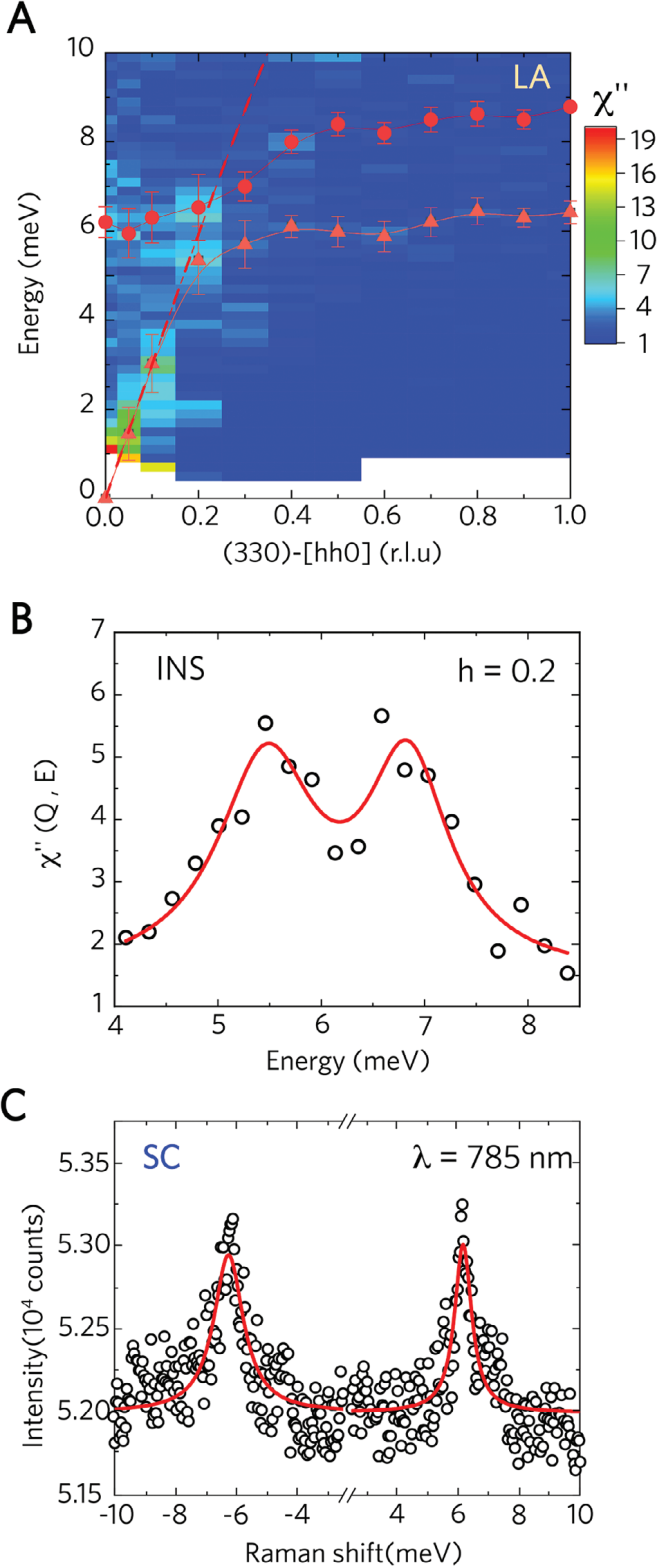
A) Phonon dispersion map *β*‐Zn_4_Sb_3_ of at 300 K, measured in the longitudinal scan along [hh0]. The dashed curve indicates the sinusoidal function. The dynamic response function is shown by the color scale. The error bars represent the full‐width‐at‐half‐maximum of the phonon peaks. B) The constant‐Q scan at (330) – [0.2 0.2 0] near the crossing point, shows two well‐define peaks. C) Raman spectrum of the SC sample at room temperature, excited at a wavelength of 785 nm. The solid lines in (A) and (C) are the Lorentzian fits to the data.

Our inelastic neutron scattering (INS) results offer direct insight into phonon behavior, shedding light on the origins of low *κ*_L_ in *β*‐Zn₄Sb₃ single crystals. **Figure** **3**A presents the dispersions of the longitudinal phonon modes along the [*hh*0] direction near the (330) reciprocal lattice vector, as measured by inelastic cold neutron scattering. An avoided crossing between the longitudinal acoustic (LA) phonons and a flat rattler mode is observed, where the emergence of the low‐energy rattler mode softens the LA phonons. Two scattering peaks are detected in the energy scan at ℎ = 0.2 near the avoided crossing point, as shown in Figure 3B. The flattening of the LA mode at large wavevectors deviates from harmonic dispersion (dashed line in Figure 3A), indicating strong anharmonicity due to significant phonon‐rattler scattering. The presence of the rattler mode is further confirmed by Raman scattering, with both Stokes and anti‐Stokes peaks observed at 6.17(2) and −6.26(3) meV at room temperature, as shown in Figure 3C. These peaks indicate a rattler mode energy of 6.2 meV (50 cm⁻¹) at the zone center.

Since acoustic phonons primarily influence *κ*_L_, the flattened LA dispersion by avoiding crossing suggests a small group velocity, contributing to low thermal conductivity. The group velocity is defined as the derivative of the angular frequency with respect to the wavevector, ∂ω /∂q. The LA phonon group velocity near ℎ = 0 is 4222 m s^−1^, slightly higher than the 4096 m s^−1^ observed in Ba_8_Ga_16_Ge_30_,^[^ ^10^ ^]^ which also exhibits an avoided crossing. Based on the harmonic dispersion in Figure 3A, the average group velocity is estimated to be 4152 m s^−1^ between ℎ = 0.2 and 0.3. However, our INS results show a remarkable reduction in this range, with the average group velocity dropping to 591 m s^−1^ due to the flattening of the dispersion resulting from the avoided crossing. The LA phonons potentially contribute more to heat transfer than the transverse acoustic (TA) phonons due to their higher group velocity. The significant reduction in LA group velocity due to the avoided crossing may be a key mechanism for the ultralow thermal conductivity in *β*‐Zn₄Sb₃. Additionally, the phonon lifetimes *τ*, derived from the full‐width‐at‐half‐maximum of the phonon peaks, are as short as 0.86 and 0.89 picoseconds (ps) for the LA phonon and rattler modes at ℎ = 0.2, respectively. These lifetimes are shorter than the 1.3 and 1.5 ps observed for Ba₈Ga₁₆Ge₃₀ near the crossing point.^[^ ^10^ ^]^ For ideal harmonic phonons, which are non‐interacting, have infinite phonon lifetime. The limited phonon lifetimes are attributed to phonon scattering, where phonons vibrate for a short period before being scattered by other quasiparticles, such as through electron‐phonon and phonon–phonon scattering. Additionally, scattering by the rattler atoms can further shorten phonon lifetimes. The avoided crossing in Zn₄Sb₃ caused by phonon‐rattler scattering results in phonon lifetimes less than 1 ps and an ultralow group velocity of 591 m s^−1^ near the crossing point, which accounts for the ultralow *κ*_L_. Unfortunately, confirming the existence of the avoided crossing in Poly Zn₄Sb₃ is difficult because the INS measurements for the polycrystalline sample cannot distinguish phonons propagating along the *h*, *k*, and *l* directions.

**Figure 4:**
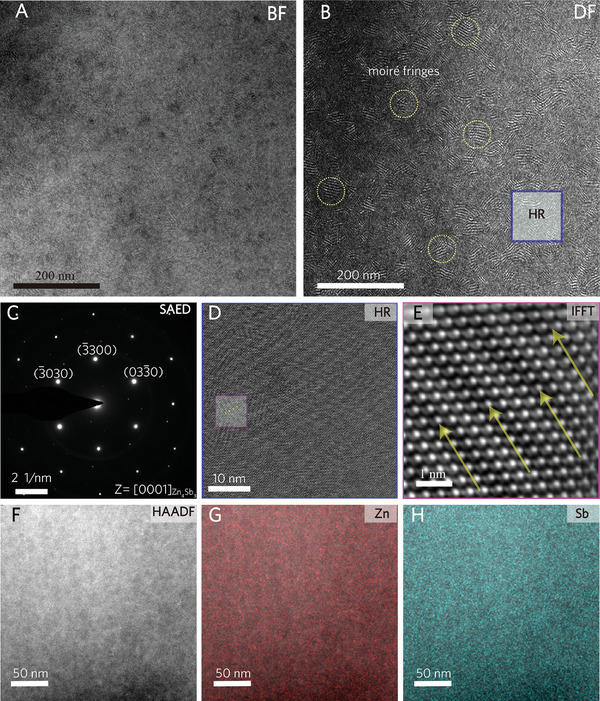
TEM characterizations of the SC sample. A) bright‐field (BF) images, B) dark‐field (DF) image, C) selected area electron diffraction (SAED) pattern, D) HRTEM image, E) IFFT image, F) STEM image, and G,H) the elemental mapping combined with HAADF image.

Given that the SC sample exhibits intrinsic and ultralow *κ*_L_, the presence of atomic‐scale defects is of critical importance. TEM characterizations of the SC sample, summarized in **Figure** **4**, reveal detailed structural and defect information. The Bright‐field (BF) image in Figure 4A shows a grain‐boundary‐free structure embedded with uniformly distributed defects on the scale of tens of nanometers. The selected area electron diffraction (SAED) pattern, displayed in Figure 4C, reveals a [0001]_Zn₄Sb₃_ zone axis corresponding to the hexagonal structure and extra‐spots created by double diffraction. The dark‐field (DF) image in Figure 4B indicates a high density of moiré fringes, suggesting localized structural imperfections. Since the sample is a single‐crystalline Zn₄Sb₃ without doping, and given that Sb atoms form a rigid framework,^[^ ^17^ ^]^ the observed moiré fringes may be attributed to the high mobility of Zn atoms. Closer examination using the high‐resolution (HR) image in Figure 4D and the enlarged atomic‐scale view in Figure 4E confirms the coexistence of well‐crystallized regions and areas with moiré fringes. This suggests that the moiré fringes originate from the overlap of a perfect hexagonal crystal with regions of Zn enrichment or Zn deficiency. The high density of these moiré fringes contributes to significant phonon scattering, thereby leading to intrinsically low *κ*_L_. Despite these imperfections being localized to tens of nanometers, the STEM image and corresponding elemental mapping in Figure 4F–H reveal a uniform distribution of Zn and Sb elements, consistent with the single‐phase and single‐crystalline nature of the material.

**Figure 5:**
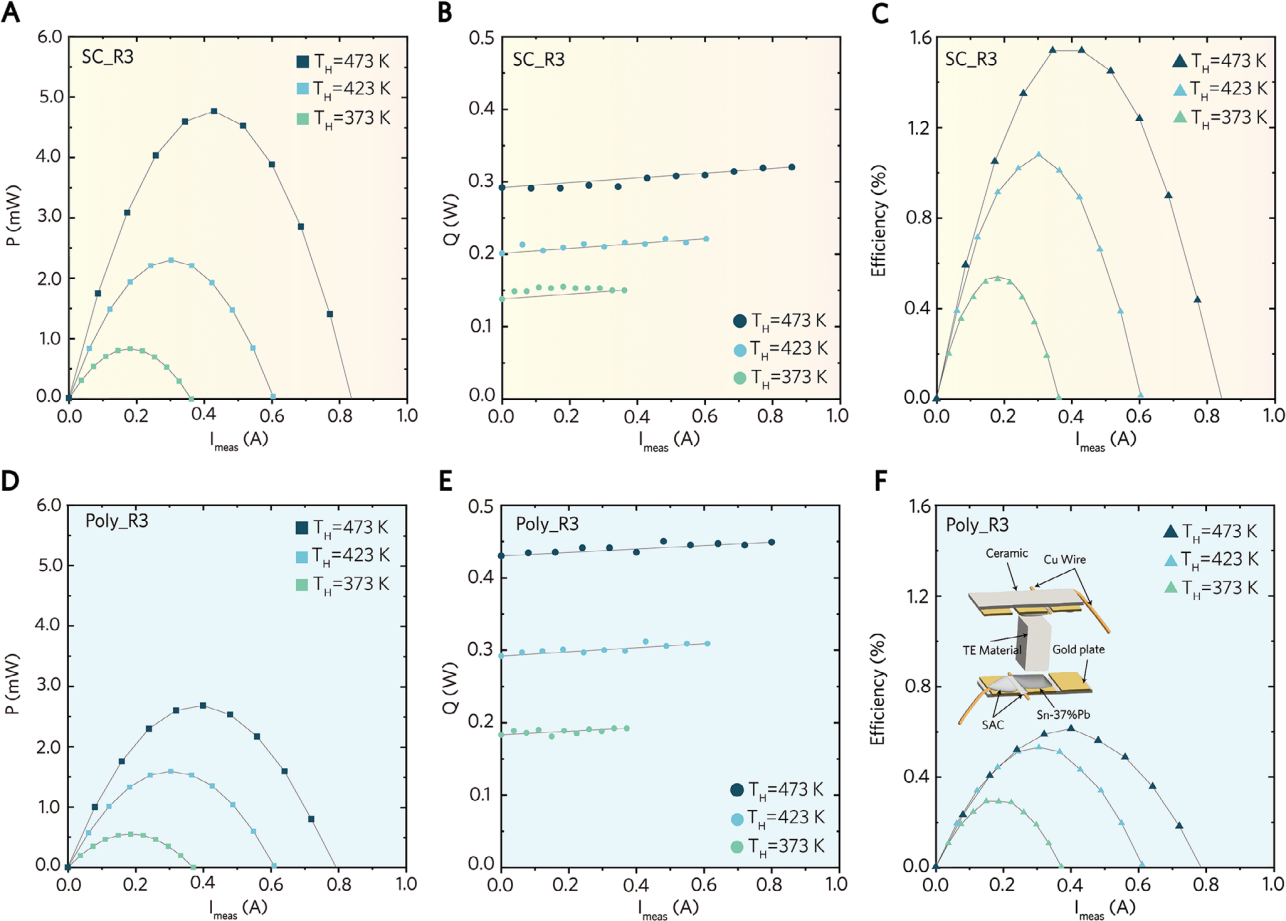
A,D) output power, B,E) heat flow, and C,F) conversion efficiency of the SC single‐leg and the Poly single‐leg respectively, as a function of electrical current at the hot‐side temperatures of 373, 423, and 473 K.

While most reports discuss the differences in the transport properties between doped and undoped Zn₄Sb₃, the conversion efficiency *η* of Zn₄Sb₃‐based systems has not yet been reported. In this study, we assembled several *p*‐type single‐leg, each utilizing either SC or Poly Zn₄Sb₃ as the TE material. Two SC single‐leg devices were assembled: one with the single‐crystalline Zn₄Sb₃ (the SC sample) and the other with poly‐crystalline Zn₄Sb₃ (the Poly sample). **Figure** **5**A–C show the output power *P* and the heat flow *Q* for the SC single‐leg (filled symbols) and Poly single‐leg (open symbols), measured by a commercial apparatus within a hot side temperature T_H_ ranging from 323 to 473 K. Both *P* and *Q* increase with rising T_H_. The SC single‐leg exhibits higher *P* and lower *Q* than the Poly single‐leg, resulting in a larger *η* under identical operational conditions and chemical composition. The higher *P* of the SC single leg, as shown in Figure 5A, is attributed to the larger power factor *PF* of the SC sample, as illustrated in Figure 2D, relative to the Poly sample. Meanwhile, the ultralow *κ*_L_ of the SC sample, as depicted in Figure 2C, accounts for the lower heat loss in the SC single leg, as shown in Figure 5B. Consequently, the peak *η* reaches 1.4% in the SC single‐leg at a T_H_ of 473 K, 165% higher than that of the Poly single‐leg, as shown in Figure 5C. The above results suggest that, even with identical chemical compositions, the crystallinity of the materials can influence the thermoelectric conversion efficiency. Better crystallinity implies a lower defect density in TE materials, resulting in improved output power when assembled into devices.

Despite the promising results of the SC sample in terms of TE performance, there are several limitations to the present work. One of the primary challenges in the fabrication of *β*‐Zn₄Sb₃‐based TE materials is achieving uniform doping and minimizing crystal defects. The XRD analysis reveals that the formation of secondary phases, as observed in the Poly sample, further highlights the difficulty in maintaining phase purity during synthesis, which directly impacts the stability when utilizing the polycrystalline Zn₄Sb₃‐based alloys into devices. To optimize the TE properties, future work should focus on improving the doping uniformity and controlling the defect density. Additionally, strategies such as grain boundary engineering and interface control can help reduce phonon scattering and enhance electrical conductivity. Tailoring the microstructure through techniques like hot pressing or spark plasma sintering may also improve the zT values by balancing the trade‐offs between electrical and thermal transport properties. Finally, in‐depth studies of the phonon scattering mechanisms, potentially involving machine‐learning‐assisted simulations, could provide further insights into how to minimize the *κ*_L_, enhancing the overall efficiency of these materials.

The present results underscore the potential of tellurium‐free thermoelectric materials, such as Ag_2_Se‐based alloys^[^ ^25^, ^26^, ^27^ ^]^ and Mg_3_Sb_2_,^[^ ^28^, ^29^, ^30^ ^]^ as highly efficient and environmentally sustainable alternatives for thermoelectric generators. These tellurium‐free materials not only offer superior TE performance but also address critical concerns regarding the scarcity and toxicity of tellurium. By combining high conversion efficiency with reduced environmental impact, Ag_2_Se‐based alloys and Mg_3_Sb_2_ present promising solutions for large‐scale energy harvesting applications, potentially paving the way for more sustainable energy technologies in the future.

## 3 Conclusion

In summary, we investigated the TE properties of *β*‐Zn_4_Sb_3_ single crystals, shedding light on the mechanisms that drive their exceptional performance. Through inelastic neutron scattering (INS), we have unveiled critical insights into phonon behaviors, particularly the phonon avoided crossing, which plays a pivotal role in achieving ultralow *κ*_L_. Our research emphasizes the crucial role of crystallinity in enhancing TE efficiency, with single‐crystalline *β*‐Zn_4_Sb_3_ outperforming polycrystalline counterparts in both *zT* value and *η*. Furthermore, the single‐crystalline *β*‐Zn₄Sb₃ single‐leg exhibited a higher output power than the polycrystalline one and demonstrated an impressive conversion efficiency of 1.4% under a temperature gradient of 200 K, marking the highest efficiency reported for the Zn₄Sb₃ without dopants. These findings pave the way for developing high‐performance, tellurium‐free thermoelectric materials suitable for practical applications.

## Conflict of Interest

The authors declare no conflict of interest.

## Author Contributions

I.L.J. synthesized the samples and carried out the characterizations and thermoelectric properties measurements. I.L.J., C.Y.L., C.M.W., and C.H.L. conducted the INS measurements, while C.Y.L. and C.H.L. performed the Raman analysis. All authors contributed to the discussion. I.L.J., C.H.L., and H.J.W. contributed to the manuscript writing.

## Supporting information



Supporting Information

## Data Availability

The data that support the findings of this study are available in the supplementary material of this article.
