# Supplementary material for: Avoided Crossing Phonons Realizes High‐Performance Single‐Crystalline β‐Zn4Sb3 Thermoelectrics
Source: Adv Sci (Weinh). 2024 Dec 11;12(5):2411498. doi: 10.1002/advs.202411498 (PMC11791943; doi:10.1002/advs.202411498)
Supplement: Supplementary file 1 — Supporting Information [file ADVS-12-2411498-s001.docx]

Supporting Information

**Avoided Crossing Phonons Realizes High-Performance Single-crystalline *β*-Zn_4_Sb_3_ Thermoelectrics**

I-Lun Jen,^a,b^ Cheng-Yen Lin,^c^ Kuang-Kuo Wang,^d^ Chun-Ming Wu,^e^ Chi-Hung Lee,^c*^ and Hsin-Jay Wu^a,b*^

**Experimental Section**

(i) Synthesis:

High-purity Zn (99.99%, Alfa Aesar), Sb (99.99%, Alfa Aesar) were weighed according to the stoichiometric ratio of Zn₅₇Sb₄₃. These elements were placed in a quartz tube with an inner diameter of 7 mm, which was then sealed under vacuum using an oil-free dry scroll vacuum pump (Edwards nXDS10i). The sealed tubes were heated to 1023 K for 8 hours, held at that temperature for 14 hours, and then rapidly quenched in water to room temperature. To prevent issues related to the thermal expansion of Zn₄Sb₃, the samples were sealed in a double quartz tube with an inner diameter of 10 mm. Subsequently, a portion of the quenched sample was grown using a vertical Bridgman technique at 873 K with a growth rate of 2.3 mm/hour (the single crystalline sample), while another portion was subjected to a five-day cooling process in the furnace (the polycrystalline sample). The as-grown and annealed alloys were cut and mechanically polished for subsequent transport property measurements.

(ii) Structure characterization:

The as-grow and annealed samples were grounded by a series of SiC papers ranging from #400 to #4000. After grounding, the samples were analyzed via in-house X-ray diffraction (Bruker D2-Phaser, XRD) with Cu Kα radiation (λ = 1.5406 Å). The measurements were collected in the 2*θ* range of 15-65° with a step size of 0.01°. The in-situ synchoron-radiation powder X-ray analysis at the TPS-19A beamline of the National Synchrotron Radiation Research Center (NSRRC) in Taiwan, with the wavelength of 0.61992 Å.

(iii) TE properties measurements:

The Seebeck coefficients *S* and electrical conductivity *σ* of the samples were measured by commercial instrument (ZEM-3, ULVAC). The carrier concentration ($n_{\mu}$) and mobility ($\mu_{H}$) were obtained by Hall measurement under a magnetic field of 0.49T (HMS-3000, ECOPIA). In the thermal properties, the thermal conductivity *κ* was calculated by the formula $\kappa= D\times C_{p}\times\rho$, where *D*, measured by the light flash method (LFA 467, Netzsch), represents the thermal diffusivity, *Cp* represents theoretical heat capacity, and *ρ* represents the experimental density of the sample. The relative densities of all these pellets, measured by Archimedes method (Chrom Tech, JA-2003J), are above 98.8%.^[3]^ In addition, the electronic thermal conductivity *κ_e_* was calculated using the Wiedemann−Franz relationship *κ_e_* = *L*σT. In this equation, L represents the Lorenz number that correlates with the Seebeck coefficient via the equation $L=\left\{ 1.5+ e^{\left[ -\frac{\left| S \right|}{116} \right]} \right\}\times{10}^{-8} V^{2}K^{-2}$^[31]^ and the lattice thermal conductivity (κ*_L_*) was calculated by the formula $\kappa_{L}=\kappa-\kappa_{e}$.

(iv) TE conversion efficiency measurement*:*

The bulk samples were shaped into rectangular dimensions of 4.0 mm (depth) × 3.0 mm (width) × 7.0 mm (height). Copper wires with a diameter of 0.45 mm were used as conducting leads. During the assembly process, two types of solder paste were utilized: Sn-3Ag-0.5Cu (218°C, SAC) and Sn-37Pb (183°C). First, the copper wires were soldered to the electrodes using SAC. Next, the electrodes were connected to the single-leg thermoelectric device using Sn-37Pb solder. The single-leg device was placed into a commercially available thermoelectric conversion efficiency instrument (Mini-PEM, ULVAC). To minimize thermal contact resistance between the copper blocks and electrodes, a 200 µm thick graphite sheet (Grafoil) was applied to the hot and cold sides with thermal grease (KS-613, Shin-Etsu Silicone). The electrical current generated by the single-leg device and its conversion efficiency were measured using the Mini-PEM. During these measurements, the hot side temperature was varied from room temperature to 473 K, while the cold side temperature was maintained at 298 K.

(v) Inelastic Neutron Scattering Measurement:

The INS technique allows us to discover the phonon dispersion relation by mapping the dynamic response function χ’’(*Q*, *E*), which is the measured neutron scattering intensity divided by the Bose factor, to study the microscopic dynamics of materials, to study the microscopic dynamics of materials. In this study, the beamline SIKA—the multiplexing cold-neutron triple-axis spectrometer at ANSTO (Australia) which is applied to perform the χ’’(*Q*, *E*), where *E* is the energy transfer, and *Q* = *G* + *q* is the wave-vector transfer, with the phonon propagating vector *q* and a reciprocal lattice vector *G*. The final energy was fixed at 8.07 meV.

(vi) TEM characterization

The TEM characterization is further conducted on the SC sample, using the field-emission transmission electron microscopy (FE TEM, Thermo Fisher Scientific Talos F200X G2, 200 kV) equipped with energy dispersive X-ray spectroscopy (EDS, Bruker Dual-X), to give an insight on nanoscale feature. The cross-sectional TEM samples are prepared by the focus ion beam (FIB, Hitachi NX2000). The selected area electron diffraction (SAED), bright field (BF) image, and dark field (DF) image of TEM were used to analyze the microstructure and high-resolution (HR) lattice image analysis were performed at magnifications of 650 kx-1.05 Mx. Furthermore, the high-angle annular dark-field (HAADF) images and corresponding EDS mapping images of SC ample were obtained in the scanning transmission electron microscopy (STEM) mode.


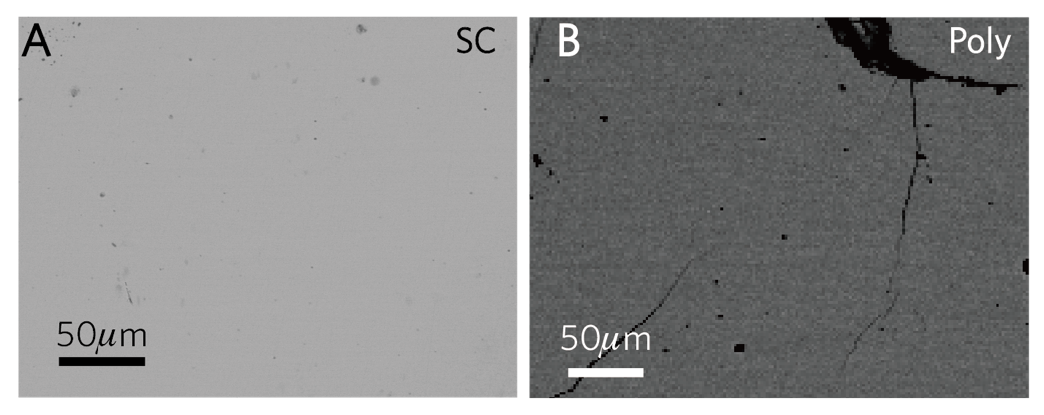


**Figure S1**. BEI images of the (a) Bridgman-grown crystal and (b) the furnace-cooled sample.


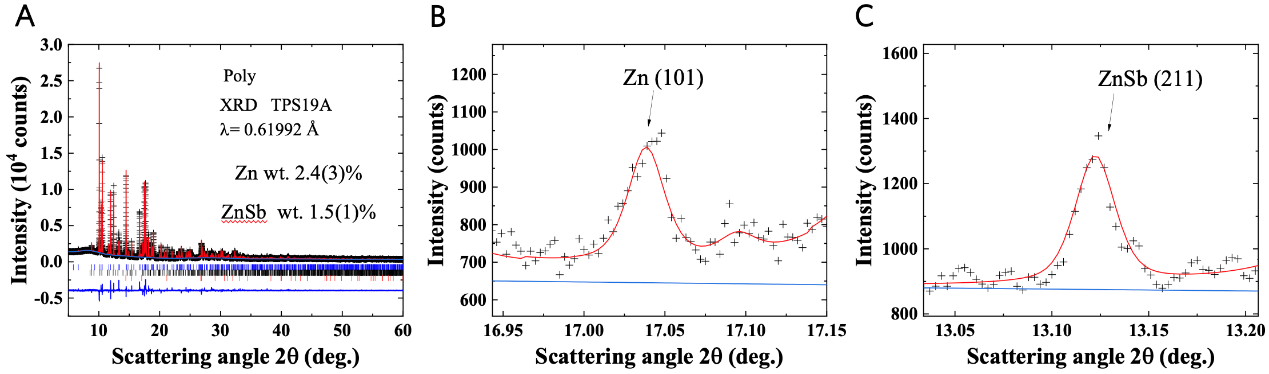


**Figure S2** The PXRD of the Poly sample. The refinement results indicate the presence of secondary phases.
